# Plateau pika fecal microbiota transplantation ameliorates inflammatory bowel disease manifestations in a mouse model of colitis

**DOI:** 10.3389/fmicb.2023.1228778

**Published:** 2023-09-19

**Authors:** Yayuan Yang, Baiqiang Cui, Yanan Lv, Xiangnan Lu, Wenxiang Shen, Min Feng, Xuezhi Ding, Pengcheng Dong, Yu Wang

**Affiliations:** ^1^Key Laboratory of Veterinary Pharmaceutical Development, Ministry of Agricultural and Rural Affairs, Lanzhou Institute of Husbandry and Pharmaceutical Sciences of Chinese Academy of Agricultural Sciences, Lanzhou, China; ^2^Department of Thoracic Surgery, Gansu Province Hospital, Lanzhou, China; ^3^College of Food Science and Engineering, Gansu Agricultural University, Lanzhou, Gansu, China

**Keywords:** inflammatory bowel disease, Plateau pika feces, fecal microbiota transplantation, gut microbiota, colitis murine model

## Abstract

Inflammatory bowel disease (IBD) is a serious global public health concern. Although the pathogenesis of the disease is currently unknown, it has been reported to be associated with both intestinal microbiota and inflammatory mediators. There is evidence suggesting that the feces of the Plateau pika is useful for treating gastrointestinal injuries and pain. Although fecal microbiota transplantation is highly efficacious intervention for IBD prevention, however, potential the transfer of pathogenic microbes or toxic substances is potentially hazardous. Fortunately, micropore filtering of the donor feces can minimize the risk of bacterial infection allowing retention of the therapeutic effects of the residual bacteriophages. Here, we demonstrated that Plateau pika feces not only alleviated the IBD symptoms but also promoted optimal structure and composition of the intestinal microbiota. Additionally, Plateau pika feces transfer also enhanced phenotypic features, such as, body-weight, disease activity index, and histological scores. In conclusion, Plateau pika feces was found to protect mice against colitis induced by dextran sodium sulfate by reducing inflammation and regulating microbial dysbiosis. These findings suggest the potential of Plateau pika feces as an alternative therapy for IBD.

## Introduction

Inflammatory bowel disease (IBD) is a progressive immune-associated disease, characterized by persistent remission and relapse, that increases the risk of colorectal cancer (Cleynen et al., 2016). The incidence of the disease is currently increasing, and it is particularly common in recently industrialized countries (Gecse and Vermeire, 2018; Xiao et al., 2019). The risk factors of IBD include dysregulation of the mucosal immune response against bacteria-derived antigens, leading to the increased production of pro-inflammatory cytokines (Olén et al., 2020). Considering the crucial relevance of intestinal microbiota in keeping pathogens at bay and in maintaining overall health, there is growing research interest in supporting and enhancing the healthy microbial ecosystem to achieve better physical health (Jess et al., 2012). Moreover, these interventions may also assist in relieving the clinical manifestations of ulcerative colitis (UC) and Crohn’s disease (CD). The small lagomorph the Plateau pika (*Ochotona curzoniae*) from the alpine meadows of the Tibetan Plateau is a keystone species as it is food for predators, and provides shelter for small nesting tiny birds in its burrows (Olén et al., 2020).

Despite its overall protective actions against harmful circumstances such as pathogen infection, inflammation often involves pathways that aggravate pathogenic processes, thereby increasing the vulnerability of the host to further attacks (Ng et al., 2017). Many cytokines, such as, TNF-α, IL-6, and IL-1 are targets of the NF-кB pathway (Nadeem et al., 2020). While inhibitor proteins (IBs) typically restrict the activity of NF-кB in the cytoplasm, after translocation of the protein to the nucleus NF-кB promotes a series of complex phosphorylation and degradation events leading to the expression of downstream target genes and subsequent pro-inflammatory signaling (Kuipers et al., 2015). The NF-кB axis is reported to be closely associated with the pathogenesis of both UC and CD in humans and animals (Odenwald and Turner, 2017; Schirmer et al., 2019).

The interaction between intestinal microbiota and pro-inflammatory factors in UC is complex (Xavier and Podolsky, 2007). Prior investigations have revealed that the alleviation of UC symptoms occurs via an intricate balance between beneficial bacteria, host genetic factors, and common environmental stimuli. The recently introduced technique of fecal microbiota transplantation (FMT) has been shown to effectively enhance the structural regulation of the intestinal microbiota, thus relieving IBD and persistent gastrointestinal dysbiosis (Roda et al., 2016; Na and Moon, 2019). Nevertheless, the precise mechanism where FMT treatment relieves UC symptoms remains undetermined, and the majority of published information to date has been from case reports and allied research.

Previous studies have shown a reduced risk of UC when Plateau pika feces, host genetic factors, and common environmental stresses interact in a balanced manner (Paramsothy et al., 2019). Here, we explored the role of Plateau pika FMT in relieving IBD in a mouse model of colitis.

## Materials and methods

### Animals

All protocols involving the C57BL/6 J mice followed the Care and Use of Laboratory Animals (Gansu Province Animal Care Committee, Lanzhou, China) guidelines, and the study received ethical approval from the Lanzhou Institute of Husbandry and Pharmaceutical Sciences, CAAS. The mice were housed in an environment with constant temperature (20–24°C) and humidity and a 12-h / 12-h light/ dark cycle. Mice received food and water *ad libitum*.

**Figure 1:**
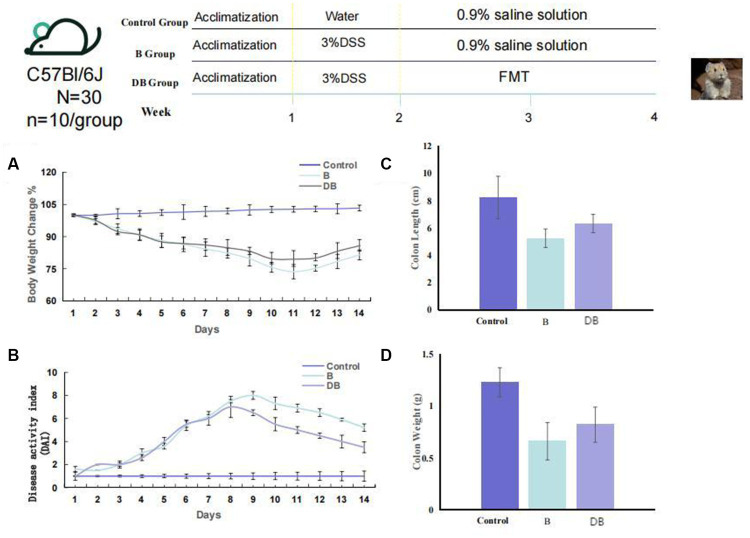
FMT reduced colonic damage of the colitis mouse model. Experimental design for FMT treatment on DSS-induced colitis in mice. **(A)** Weight loss (*n* = 10). **(B)** DAI (*n* = 6). **(C)** Colon length, and **(D)** Colon weight. Statistics were calculated with a two-tailed Student’s t test. *, *p* < 0.01; **, *p*, 0.01 **(C,D)**. Data are presented as the mean 6 SEM.

The animal protocol is presented in Figure 1. Briefly, mice(*n* = 10/ group) were randomly assigned to one of three groups: (i) control; (ii) dextran sodium sulfate (DSS); (iii) FMT. For the first 7 days, all mice, apart from the controls received drinking water containing 3% DSS solution thus establishing the UC model. Subsequently, we collected, accumulated, gently homogenized, and froze the cecal contents of 10 Plateau pikas in 10% sterile glycerol. To generate a working FMT solution, the frozen fecal material was thawed and diluted to 0.05 g/ml with sterile saline, followed by homogenization, centrifugation (5,000 g 30 min, 4°C), and subsequent filtration of the supernatant using a 0.45 μm PES filter (Minisart^®^ High Flow Syringe Filter, Sartorius^™^, Germany; Slack et al., 2022). The mice received FMT via a gavage of 200 μl of the supernatant from the Plateau pika fecal samples once a day over 2 weeks. Meanwhile, the control and DSS mice received a gavage of 200 μl of 0.9% saline solution. To establish the colitis mouse model, we supplemented sterile water with 3% DSS for 7 days, with 7 additional days of water without DSS. the pika feces supernatant was administered via gavage to the FMT mice, while control mice were given 0.9% saline solution. Daily parameters, including body-weight, changes in the consistency of the feces, and the presence of blood in the feces were monitored. To analyze the intestinal microbiota using 16S rRNA gene sequencing, feces were collected from the mice on day 14, and colonic tissue was harvested from the mice following euthanasia (Figure 1). DAI was scored as earlier described (Supplementary Table S1; Murthy et al., 1993; Camuesco et al., 2004).

After completion of the treatment, all animals were first anesthetized with sodium pentobarbital (5%), and then sacrificed by cervical dislocation immediately before dissection. Their colons opened, and the feces were gently moved using forceps. The distal colon (about 30 mm) together with additional intestinal regions were used for histological analysis using hematoxylin–eosin (HE) staining, respectively. We also collected colon samples for proinflammatory cytokine evaluation.

### Histopathological analysis and HE staining

Mouse colons were fixed in 10% buffered formalin, before paraffin embedding, sectioning into 5 μm sections, HE staining, and examination under light microscopy.

### Assessment of colonic oxidative stress (OS) parameter

Colonic parameters, namely, malondialdehyde (MDA) levels, total antioxidant capacity (T-AOC), catalase (CAT), and superoxide dismutase (SOD) activities, were measured using the appropriate kits (Nanjing Jiancheng Bioengineering Institute, China).

### 16S rRNA gene amplicon sequencing

We next determined the composition of the intestinal microbial communities in the distal ileal mucosa and intestinal lumen using 16S rRNA gene (V3-region) amplicon sequencing using NextSeq with v2 MID, 300-cycle, paired-end chemistry on a NextSeq (Illumina United States) platform. Total DNA was isolated using a Bead-Beat^®^ Micro AX Gravity Kit (A&A Biotechnology, Poland), according to the provided directions. A library was generated, as reported previously (Slack et al., 2022). The mean amplicon sequencing depth per sample was 35,840 reads (minimum 9,286; maximum 63,584 reads).

### Statistical analysis

Data were analyzed using GraphPad Prism and SPSS. *p* < 0.05 was set as the significance threshold. Data are presented as mean ± standard deviation (SD) and compared using one-way ANOVA and Duncan’s test.

## Results

### FMT-mediated regulation of mouse colitis development and clinicopathological manifestations

Among other consequences, DSS-triggered colitis causes the formation of ulcers in the intestinal epithelium, resulting in disruption of the inner mucosal layer, and promoting the invasion of acute and inflammatory immune cells (Das et al., 2020; Bell et al., 2022). Typical biomarkers of IBD are body weight, disease activity index (DAI) scoring, and the length/colon weight ratio (Ni et al., 2017; Chen et al., 2021). These variables were, therefore, used in the present study to evaluate the FMT-mediated regulation of colitis development and progression *in vivo*. It was found that all three variables were significantly reduced in DSS-treated mice (*p* < 0.01) compared with the controls (Figure 1). Significant weight gain was observed after FMT administration, which was equivalent to the weight of the control mice (*p* < 0.05). FMT-treated mice also showed substantially reduced DAI scores. Together, these results are consistent with previous findings (Weingarden and Vaughn, 2017), and indicated that treatment with FMT could successfully prevent DSS-driven IDB.

### Plateau pika feces mitigated the pathological manifestations of DSS-induced experimental UC

We developed a DSS-induced mouse model of UC using 14-week-old C57BL/6 J mice to elucidate the influences of Plateau pika microbiota transplantation on UC. Relative to the control mice, the mice receiving Plateau pika feces microbiota transplantation showed significant improvements in their colons (Figure 1). The changes in body weight of the FMT-treated mice were also moderate. It was also found that the FMT treatment strongly prevented weight loss, relative to the control mice (*p* = 0.038; Figure 1A). Additionally, the DAI scores of the FMT-treated mice were substantially reduced, in comparison with the controls (*p* = 0.027; Figure 1B).

### FMT-mediated regulation of histopathological changes in the colon

**Figure 2:**
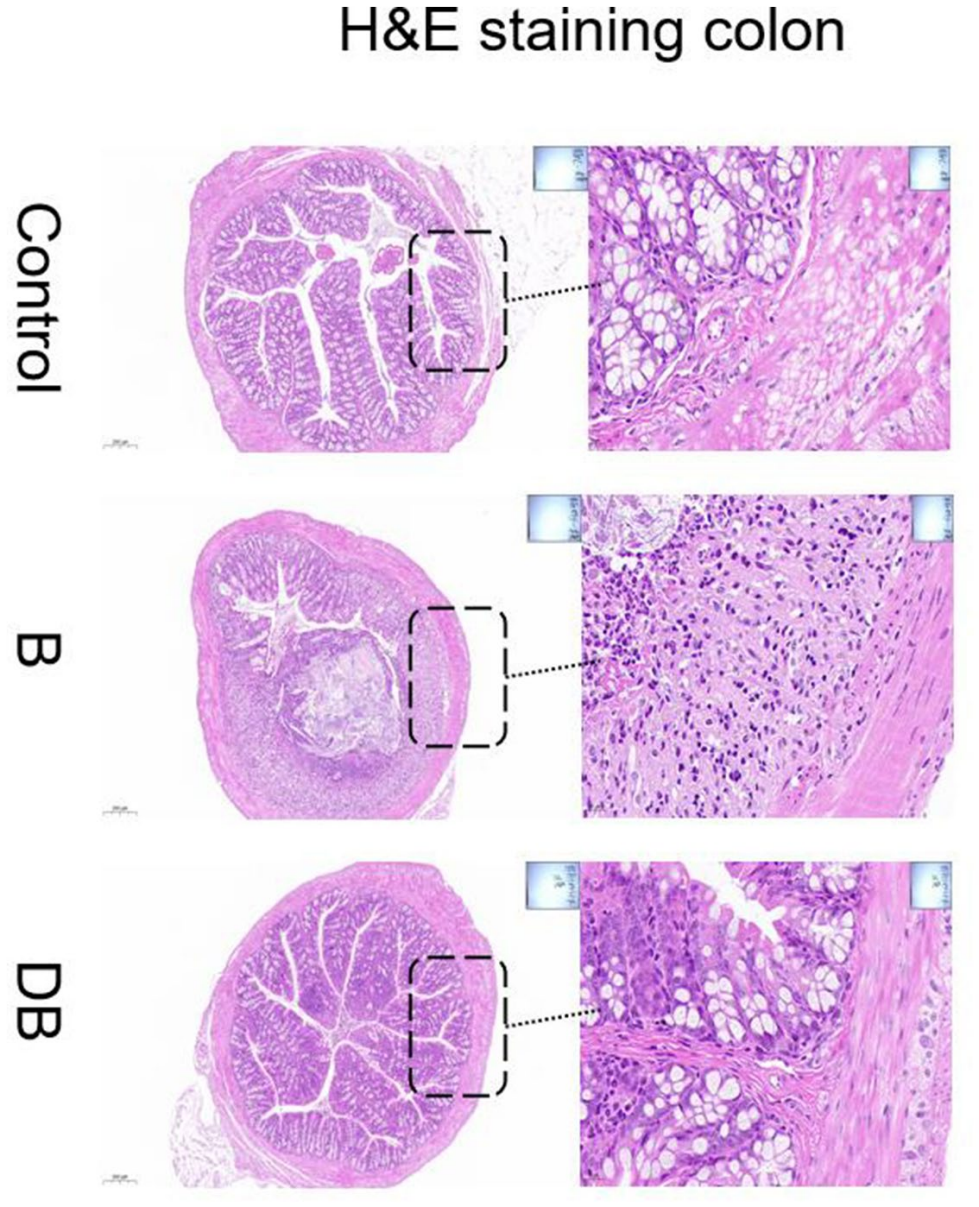
Effects of FMT intervention on the mouse colon histological changes (Representative hematoxylin and eosin staining) (Control) Control group, (B) B group, (DB) DB group.

**Figure 3:**
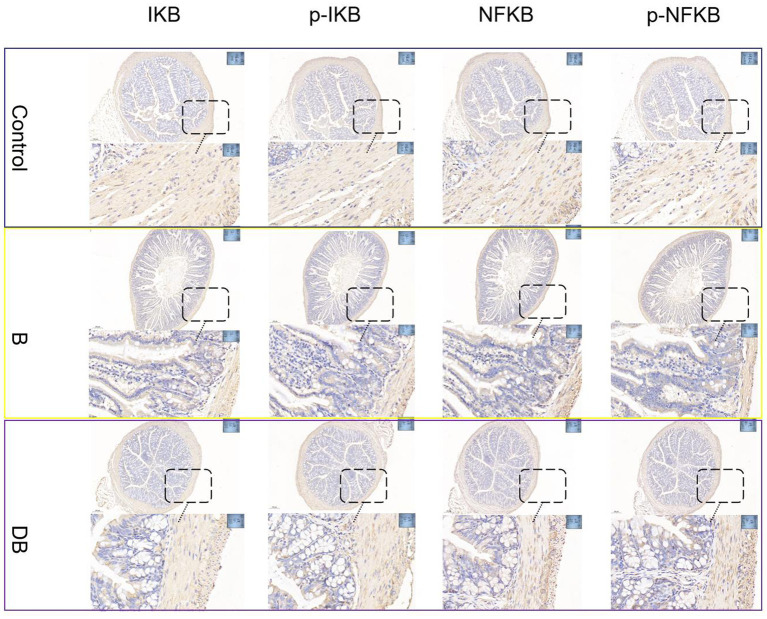
FMT modulated DSS-induced NF-κB activation in the colonic tissue. NF-κB protein levels in the colonic tissue were analyzed by immunohistochemical.

Representative images of the colon histology from each group of mice are shown in Figure 2. As expected, control mice showed normal colon histomorphology (Figure 2). In contrast, the DSS-treated mice showed thickening of the intestinal walls, submucosal edema, mucosal invasion, cytoplasmic mucin depletion, and ulceration of the colon (Figure 2). These characteristic symptoms of UC were significantly reduced after FMT administration (Figure 3). These results are consistent with those of Newman et al. (2017).

### FMT-mediated regulation of oxidative status in colonic tissue

**Figure 4:**
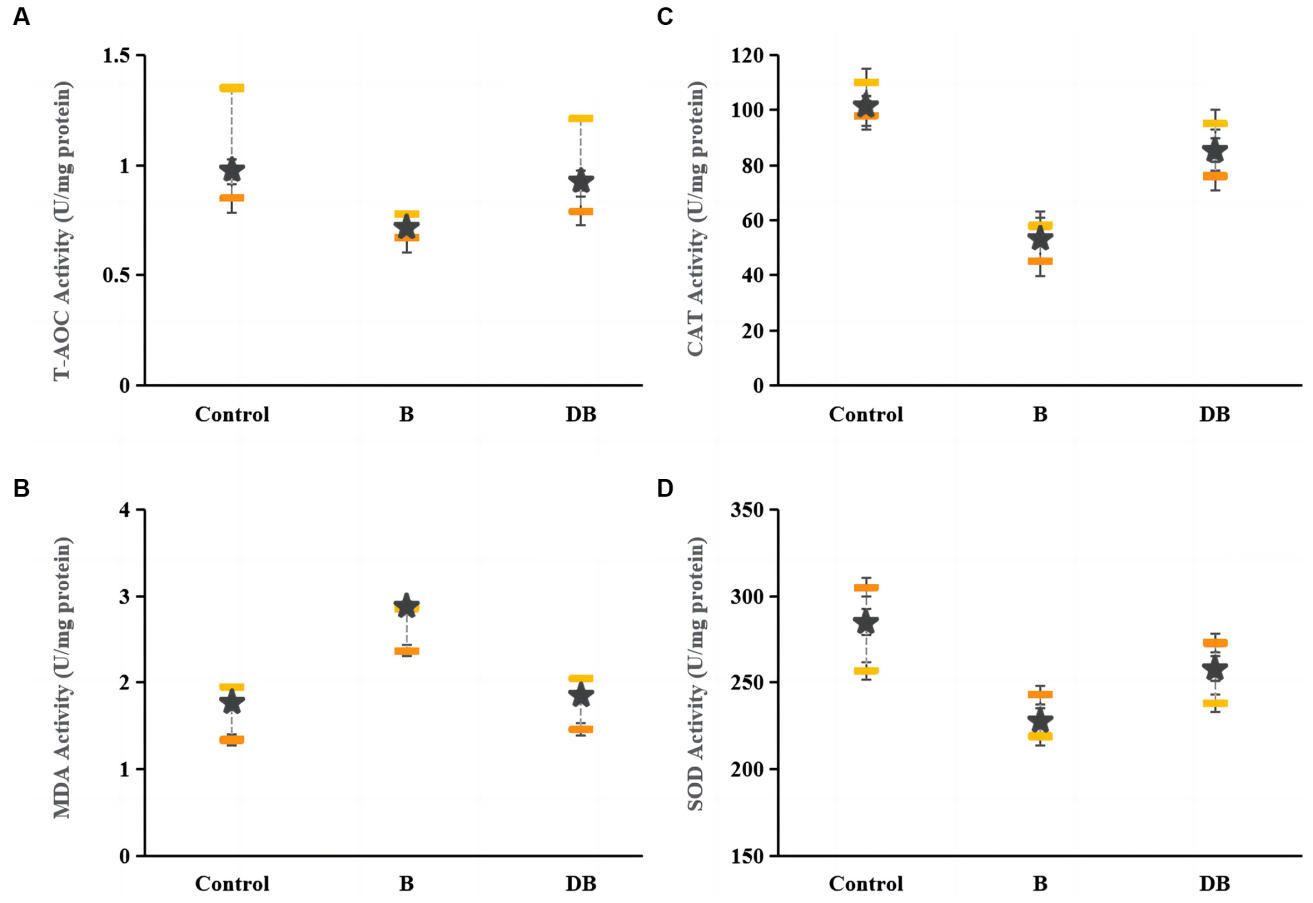
Effects of FMT intervention on colonic oxidative stress parameters in DSS-induced colitis mouse. **(A)** T-AOC, **(B)** MDA, **(C)** CAT, and **(D)** SOD. Data are presented as mean ± SD. ^##^*p* < 0.01 and ^#^*p* < 0.05 vs. the control group. **p < 0.01 and *p < 0.05 vs. the B model group.

The present as well as prior UC studies demonstrated a link between intestinal OS and disease (Figure 4). Reactive oxygen species (ROS) are typically produced when proinflammatory factors activate phagocytes (Sun et al., 2017). Hence, any intervention that enhances the cellular antioxidant capacity can potentially prevent the development and severity of UC (Paramsothy et al., 2017; Mishra et al., 2022). Markedly reduced levels of CAT, SOD, and T-AOC together with significantly raised MDA levels were observed in DSS mice relative to the controls. CAT requires the splitting of the superoxide anion to catalyze O_2_ reduction to H_2_O_2_, as well as H_2_O_2_ conversion to water (Ortiz-Rivera et al., 2017), while T-AOC modulates body function and the susceptibility to certain diseases (Özçam et al., 2019). The MDA content is a well-established indicator of OS (Özçam et al., 2022). This evidence suggests that the colon undergoes OS during UC. Here, it was found that after FMT treatment, the CAT, SOD, and T-AOC levels rose significantly (*p* < 0.05) while those of MDA declined sharply (*p* < 0.01), thus indicating that FMT treatment could successfully reduce OS.

### Plateau pika feces modulated the composition of the intestinal microbiota in a DSS-induced mouse model of UC

**Figure 5:**
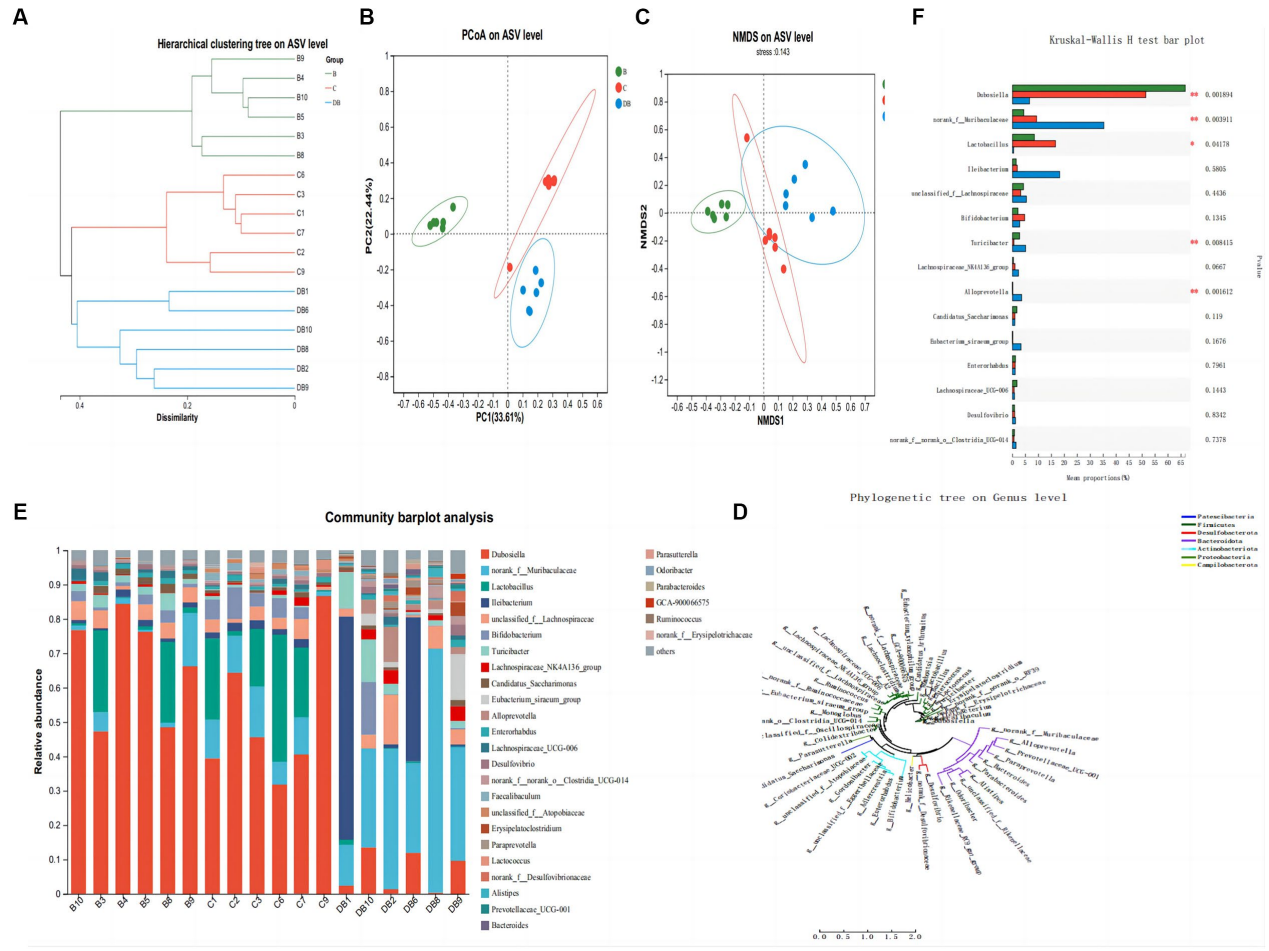
Influences of FMT administration on gut microbiota β-diversity. **(A)** Hierarchical clustering tree of weighted UniFrac distance, and **(B)** Principal coordinates analysis (PCoA) of ASV level. **(C)** NMDS of ASV level. **(D)** Phylogenetic tree on genus level. **(E)** Community barplot analysis. **(F)** Kruskal-Wallis H test bar plot. Data are presented as mean ± SD. ^##^*p* < 0.01 and ^#^*p* < 0.05 vs. the control group. **p < 0.01 and *p < 0.05 vs. the DSS model group.

Colitis is closely linked to modifications in the composition and organization of the intestinal microbiota (Sartor and Wu, 2017). We used 16S rRNA sequencing of fecal samples collected after 2 weeks of DSS treatment to explore the significance of Plateau pika feces for regulating intestinal microbiota in a DSS-driven mouse model of colitis. The rarefaction analysis showed that the bacterial composition was quite diverse (Zhu et al., 2018). The intestinal microbiota of all three groups differed in their species compositions (Figure 5). The Simpson index was used to evaluate the alpha diversity at the species level in samples from all three groups collected on day 14. This showed that the Simpson index of FMT-treated mice differed markedly from that of the control mice (Figure 5). Treatment with Plateau pika feces increased the composition and variety of the microbiota throughout the chronic inflammatory phase, which, in turn, accelerated healing. Measurement of beta diversity using principal coordinate analysis (P-CoA) with the Bray Curtis distance metric revealed two distinct clusters for the FMT and control mice (Figure 5), further indicative of significantly altered microbial compositions, even in species diversity, in the FMT-treated mice by day 14. Our overall findings revealed that FMT treatment strongly regulated the intestinal microbiota throughout the chronic and recuperation stages of intestinal inflammation. Moreover, FMT-treated mice exhibited unusual microbial compositions throughout recovery from intestinal inflammation, namely, the presence of *Dubosiella* and *Lactobacillus*, while *Ileibacterium*, unclassified_*f_Lachnospiraceae*, *Bifidobacterium*, *Turicibacter*, *Lachnospiraceae_NK4A136_group*, and *Candidatus_Saccharimonas* were more prevalent on day 14.

## Discussion

The aim of this investigation was to determine the overall effects of Plateau pika feces on recovery from intestinal inflammation in a DSS-induced mouse model of colitis. The relief of UC symptoms may have resulted from the restoration of the intestinal structure, function, and micro-environment. The findings of this study provide important information on alternative treatment methods for colonic inflammation employing functional probiotics and the use of Plateau pika feces in IBD.

We demonstrated that Plateau pika feces alleviated the symptoms of DSS-triggered colitis, shown in reduced colon length, weight-loss, DAI scores, mucosal loss, and the invasion of inflammatory cells, as documented in previous publications (Park et al., 2018; Wang et al., 2020, 2021; Jeong et al., 2022).

Importantly, the Plateau pika feces cohort outperformed single-strain mice in terms of minimizing body-weight fluctuations, DAI, and colonic histological scores (Kühl et al., 2015; Gasaly et al., 2021). This may be because, after entry into the gut, bacterial strains may directly diminish inflammation by modulating the total microbial network, apart from their interaction with mucosal epithelial cells (Liang et al., 2021). We also confirmed that on day 8 of the development of colonic inflammation and on day 14 of healing in colitis mice, Plateau pika feces effectively rescued micro-ecological dysregulation. Based upon reports, altered microbiota may prevent colitis progression. The alpha and beta diversities revealed that the structural composition of the FMT mice intestinal microbiota differed significantly from that of the controls (Sun et al., 2020; Wang et al., 2021). The single strain-based intestinal microbiota recovery mostly occurred on day 14, which made it less efficacious than the Plateau pika feces. Metabolites that enter hosts are quickly absorbed into the intestine. They also reach the small intestinal epithelial cells, whereby their utilization is further augmented.

The intestinal microbiota is modulated directly by metabolites as they include bacteriocins with significant antibacterial activity (Salminen et al., 2021). The logical conclusion is that following absorption into the gastrointestinal tract, metabolites may potentially exhibit indirect anti-inflammatory activities through additional regulation of microbial communities.

Considering such significant alterations of the microbiota in FMT-treated colitis, we consider that treatments with Plateau pika feces have great potential in treating UC. Consequently, RFCV discovered 11 unique bacterial taxa. Given that only three taxa, namely, *Lactobacillaceae Lactobacillus*, *Dubosiella Firmicutes*, and *Rikenellaceae Rikenellaceae* were strongly influenced by the single strains suggests that single strains are less beneficial for enhancing the intestinal microbiota. The Plateau pika feces are particularly controlled by the other eight taxa. it was found that six taxa, namely, *Muribaculaceae Muribaculaceae*, *Bacteroidaceae Bacteroides*, *Oscillospirales* UCG.005, *Lachnospiraceae Ruminococcus*, *Tissierellales Anaerovoracaceae*, and *Clostridia Clostridia*, were abundant in the FMT-treated group, relative to controls. *Muribaculaceae* is known for its effect in lowering inflammation, inhibiting dangerous microorganisms, and fostering anticancer immunity (Setoyama et al., 2003). Bacteroides are potent candidates for next-generation probiotics due to their unique ability to generate short-chain fatty acids and sphingolipids that support intestinal barrier integrity, as well as modulation of the immune response, which, in turn, repairs intestinal damage (Brown et al., 2019; Wang et al., 2022). The other four species produce butyrate, with *Oscillospirales* UCG.005 and *L. ruminococcus* showing a relatively low abundance in patients with UC and CRC patients (Mancabelli et al., 2017). The remaining two Clostridia taxa enhance the proliferation and differentiation of regulatory T cells to alleviate IBD and allergic diarrhea (Atarashi et al., 2013). Conversely, here, DSS treatment strongly increased the relative abundances of *Parasutterella Burkholderiales*, and *Bacteroidales Tannerellaceae* in mice, consistent with the conclusions of prior publications (Silvestri et al., 2020).

IBD patients have increased numbers of *P. burkholderiales*, which is correlated with persistent colonic inflammation (Chen et al., 2018; Zhao et al., 2021). Importantly, there are no significant variations in the relative abundances of the eight taxa in the metabolite intervention experiment between the mix and control groups, indicating that mixed metabolites cannot fully control the colonic microbiota. As a result, we hypothesized that metabolite delivery would influence microbial ecology in the small intestine. The relative abundance of *Acidiferrobacterales Acidiferrobacteraceae*, *Sphingomonadaceae Sphingosinicella*, and *Reyranellaceae Reyranella* was markedly reduced in the controls, confirming the supposition that metabolites act by balancing the small intestine microenvironment.

Despite widespread acknowledgement that IBD is correlated with alterations in the composition and metabolism of the intestinal microbiota, there have been no reports of a causal association between dysbiosis and IBD in humans (Ni et al., 2017). There is very limited research on the association between intestinal microbiota and the IBD phenotype. Here, our RDA provided strong evidence for an association between microbial and colitis phenotypic data in the probiotic FMT group, whereby core species-level microbial data provided essential phenotypic data, such as disease severity and colonic pathology. Co-occurrence networks to established to investigate the relationship between the phenotypic data and the microbiological markers. In FMT-treated mice, we clearly identified the presence of *B. bacteroides*, *M. muribaculaceae*, *B. tannerellaceae*, and *P. burkholderiales*, as well as a stronger and broader interaction network on day 8. Since many microbial markers in the metabolite intervention experiments were stand-alone taxonomic units and not symbiotic communities, they obviously did not affect phenotypic parameters. In all the interventions, *M. muribaculaceae* was found to be pivotal in the co-occurrence networks, indicating its importance in the interplay between the general microbiota and disease phenotypes. Collectively, our findings and those of other published work demonstrated that Plateau pika feces might effectively reduce intestinal inflammation and restore function in a colitis animal model.

## Conclusion

Here, we demonstrated that Plateau pika FMT relieved the intestinal inflammatory symptoms associated with colitis by altering the structure and composition of the intestinal microbiota. Our findings demonstrated a strong link between the intestinal microbiota and the colitis phenotype. These findings in an animal model highlight the potential use of Plateau pika FMT in the maintenance of intestinal health and may contribute toward the development of clinically applicable Plateau pika FMT-based interventions for IBD and other intestinal inflammatory diseases.

### Limitations of our research

The current investigation has several limitations. Firstly, we did not employ metagenomics for the evaluation of bacterial community composition. This will be done in future investigations. Secondly, to identify suitable potential therapeutic targets, the associated intestinal microbiota- and inflammation-related pathways require further examination. Lastly, additional metabolomics research is warranted to clarify the methods of the purification and identification of vital components that operate via the mixed-metabolite intervention.

#### Importance

IBD is a chronic, nonspecific inflammatory disease. It is characterized by ulceration and erosion of the colonic mucosa and can also cause a wide variety of other lesions that frequently affect the entire colon. In this study, we investigated the efficacy of Plateau pika feces administration in reducing gut inflammation and the restoration of gut microecology restoration. The results showed that Plateau pika feces significantly reduced inflammation and accelerated recovery when compared with the control group. These findings support the use of Plateau pika feces as an alternative therapeutic approach for IBD.

## Data availability statement

The data presented in the study are deposited in the NIH repository, accession number SUB13763520.

## Ethics statement

The animal study was approved by Lanzhou Institute of Husbandry and Pharmaceutical Sciences, CAAS. The study was conducted in accordance with the local legislation and institutional requirements.

## Author contributions

XD, PD, and YW designed the experiment and acquired grants. YY, BC, WS, and MF performed the experiments. YY, YL, and XL collected and analyzed the data. YY drafted and edited the manuscript. All authors contributed to the article and approved the submitted version.

## Funding

This work was supported by Gansu Provincial Youth Science Foundation Project (grant no. 22JR5RA043), The Central Level Scientific Research Institutes for Basic R & D Special Fund (no. 1610322023010), Gansu Provincial Science and Technology Plan Project (grant no. 22JR5RA039), and National Key R&D Plan (grant no. 2021YFF0702405).

## Conflict of interest

The authors declare that the research was conducted in the absence of any commercial or financial relationships that could be construed as a potential conflict of interest.

## Publisher’s note

All claims expressed in this article are solely those of the authors and do not necessarily represent those of their affiliated organizations, or those of the publisher, the editors and the reviewers. Any product that may be evaluated in this article, or claim that may be made by its manufacturer, is not guaranteed or endorsed by the publisher.
