# Supplementary material for: Plateau pika fecal microbiota transplantation ameliorates inflammatory bowel disease manifestations in a mouse model of colitis
Source: Front Microbiol. 2023 Sep 19;14:1228778. doi: 10.3389/fmicb.2023.1228778 (PMC10546031; doi:10.3389/fmicb.2023.1228778)
Supplement: Supplementary file 1 [file Table_1.DOC]

Table S1 Scoring criteria for DAI

| Score | Body weight loss | Stool consistency |
| --- | --- | --- |
| 0 | None | Normal |
| 1 | 1-5% | - |
| 2 | 5-10% | Loose stools |
| 3 | 10-20% | - |
| 4 | ＞20% | Diarrhea, gross bleeding |
